# HPV6/11 Persistence and Treatment-Associated Immune Remodeling in Recurrent Respiratory Papillomatosis: A Cross-Cohort Transcriptomic Analysis

**DOI:** 10.3390/v18101080

**Published:** 2026-09-30

**Authors:** Natalia Jędruchniewicz, Tomasz Klimek, Hanna Klimza, Krystyna Dąbrowska, Małgorzata Wierzbicka

**Affiliations:** 1 Research and Development Center, Regional Specialist Hospital in Wroclaw, 51-124 Wroclaw, Poland; tomasz.klimek@wssk.wroc.pl (T.K.); haniaklimza@gmail.com (H.K.); wierzbicka.otolaryngology@gmail.com (M.W.); 2 Laboratory of Phage Molecular Biology, Hirszfeld Institute of Immunology and Experimental Therapy, Polish Academy of Sciences, 53-114 Wroclaw, Poland; krystyna.dabrowska@hirszfeld.pl; 3 Faculty of Medicine, Wroclaw University of Science and Technology, 50-370 Wroclaw, Poland; 4 Institute of Human Genetics, Polish Academy of Sciences, 60-479 Poznan, Poland

**Keywords:** recurrent respiratory papillomatosis, human papillomavirus 6, human papillomavirus 11, viral persistence, transcriptomics, single-cell RNA sequencing, cellular immunity, TGF-β, INO-3107

## Abstract

Recurrent respiratory papillomatosis (RRP), caused mainly by human papillomavirus types 6 and 11 (HPV6/11), can persist despite the absence of recognized systemic immunodeficiency. We reanalyzed three transcriptomic datasets using the same predefined immune-gene programs: paired papilloma and autologous laryngeal mucosa from 12 patients, single-cell RNA sequencing from papillomas of four RRP donors and pharyngopalatine mucosa from three controls, and paired papilloma samples from 17 patients before and after INO-3107, a DNA immunotherapy encoding HPV6/11 antigens and interleukin-12. In untreated tissue, cluster of differentiation 8 (CD8)- and natural killer (NK)-associated cytotoxic program scores were lower and the transforming growth factor beta (TGF-β) suppressive program score was higher, reproducing findings from the same cohort. The lower cytotoxic pattern was not reproduced within the recovered combined T-cell and NK-cell population, although no single-cell comparison remained significant after correction for multiple testing. After INO-3107, CD8- and NK-associated cytotoxic, T helper 1 (Th1)/interferon gamma (IFN-γ), and C-X-C motif chemokine receptor 3 (CXCR3)-associated trafficking program scores increased significantly, whereas TGF-β and M2-like suppressive programs showed no comparable statistically supported change. Together, these findings show that different components of the local immune response do not change in parallel and support a model of selective treatment-associated immune remodeling. This pattern may inform future biomarker and combination-treatment studies.

## 1. Introduction

Recurrent respiratory papillomatosis (RRP) is a chronic disease of the respiratory epithelium caused predominantly by low-risk human papillomavirus (HPV) types 6 and 11. Lesions arise most often in the larynx and can recur repeatedly despite surgery, causing dysphonia, airway compromise, repeated anesthesia, and substantial cumulative morbidity [1,2]. Surgical removal controls the immediate airway problem but does not eliminate the underlying HPV infection.

The persistence of HPV6/11 in otherwise immunocompetent patients suggests that local immune regulation plays an important role in RRP [3]. Studies of papilloma tissue have reported reduced cytotoxic T-cell activity, a shift toward T helper 2 and regulatory T-cell responses, expression of programmed cell death protein 1 (PD-1) and programmed death-ligand 1 (PD-L1), altered antigen-presenting cell function, and suppressive chemokine signaling [4,5,6,7,8]. Transforming growth factor beta (TGF-β) produced within papillomas can also reduce natural killer (NK)-cell activity [9]. At the same time, HPV6/11-reactive T cells are still present in papilloma tissue. This suggests that viral persistence reflects incomplete local immune control rather than a complete absence of antiviral lymphocytes [10,11,12,13].

Clinical studies also support an important role for cellular immunity in RRP. PD-L1 blockade has reduced disease burden in some patients and has been associated with HPV-specific T-cell activity within papillomas [14]. Prophylactic HPV vaccination, when used therapeutically, has also been associated with fewer procedures in retrospective RRP cohorts [15]. More recently, HPV6/11-directed immunotherapies have shown encouraging clinical effects. PRGN-2012 (zopapogene imadenovec-drba; Papzimeos), a non-replicating adenoviral-vector immunotherapy encoding HPV6/11 antigens, reduced the need for surgery in adults and was approved by the US Food and Drug Administration in 2025 [16,17]. INO-3107 is a DNA immunotherapy encoding HPV6/11 E6/E7 antigens together with interleukin-12 and is administered intramuscularly with electroporation. It has been associated with fewer surgical procedures, expansion of HPV6/11-reactive T cells, and movement of treatment-induced T-cell clones into airway lesions [18,19,20].

An important question remains: how are the immune abnormalities seen in untreated papilloma related to the cellular context of the tissue, and do treatment-associated immune changes involve the same components of local immunity? A lower cluster of differentiation 8 (CD8)- or NK-associated cytotoxic program score in whole tissue could reflect differences in immune-cell abundance, subset composition, tissue localization, gene expression within the recovered cells, or a combination of these factors. Whole-tissue data alone cannot distinguish between these possibilities. At the same time, it remains unclear whether the immune programs that change after HPV6/11-directed immunotherapy mirror those that differ in untreated papilloma or represent a more selective immune response.

We therefore reanalyzed three public transcriptomic datasets representing untreated paired tissue, single-cell data, and paired tissue collected before and after HPV6/11-directed immunotherapy, applying the same predefined immune programs while keeping the statistical analyses appropriate to each dataset separate. We asked whether the patterns seen in whole tissue were also present within recovered immune-cell groups, whether the proportions of recovered cell types differed between RRP and control samples, and whether treatment-associated changes involved the same immune programs that differed in untreated papilloma. The single-cell analyses were exploratory and were not intended to establish a cellular mechanism of disease persistence.

## 2. Materials and Methods

### 2.1. Study Design and Public Datasets

This study reanalyzed three publicly available transcriptomic datasets. We refer to the paired untreated tissue analysis as P1, the donor-level single-cell expression analysis as P2, the recovered-cell composition analysis from the same single-cell dataset as S2, and the paired pre- and post-treatment analysis as P3.

**Figure 1:**
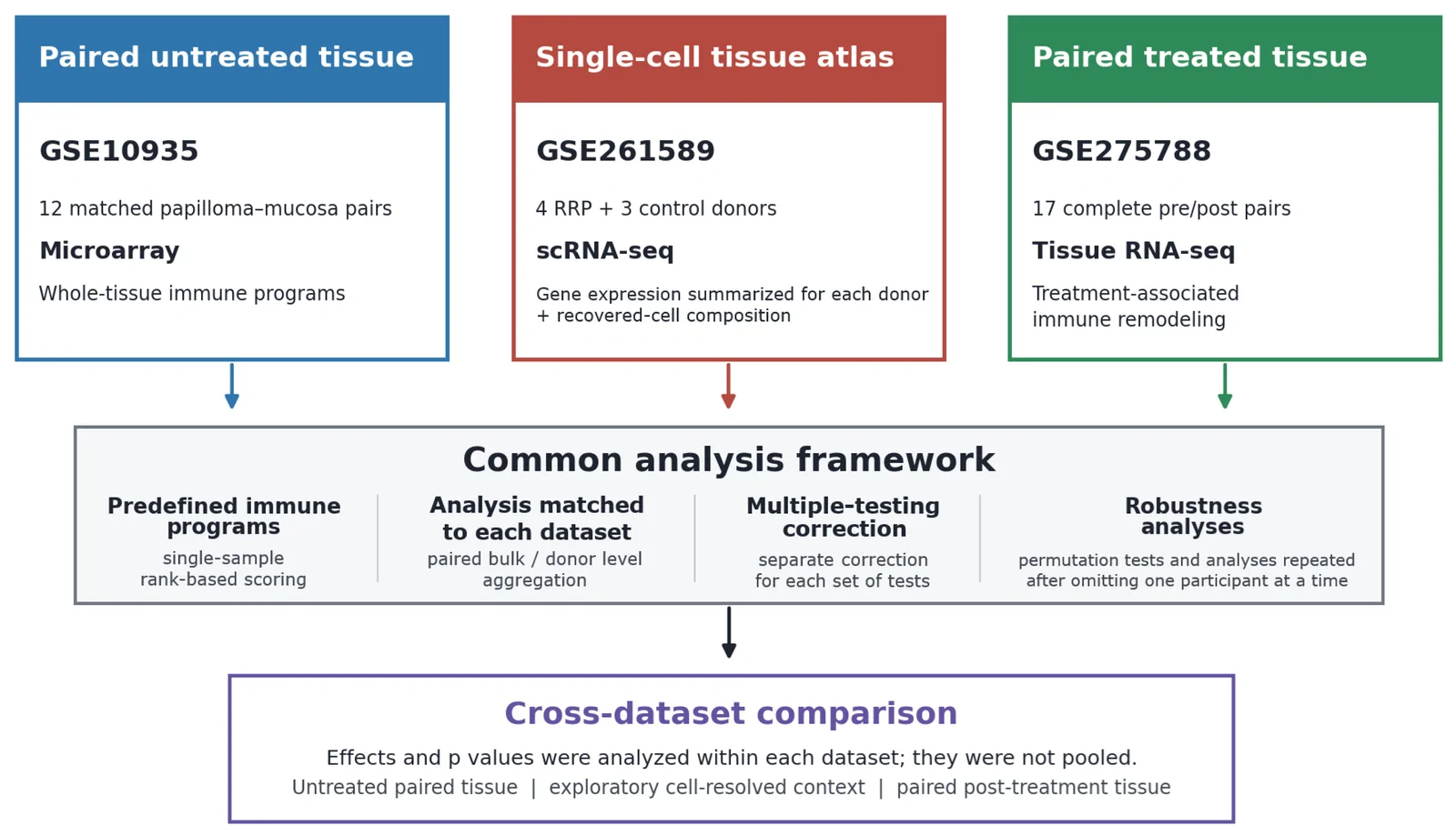
Study design and analytical framework. GSE10935 provided paired untreated papilloma and autologous laryngeal mucosa; GSE261589 provided single-cell data for exploratory donor-level cell-compartment expression and recovered-cell composition; and GSE275788 provided paired papilloma tissue before and after INO-3107. A common predefined immune program framework was applied while keeping statistical analyses separate and appropriate to each dataset. P2/S2 findings were prespecified as exploratory and were not treated as confirmatory evidence.

Before examining the results, we defined the immune programs to be tested, the expected direction of each effect, the minimum number of genes required for analysis, the approach to multiple-testing correction, the quality control criteria, and the planned sensitivity analyses. Figure 1 and Table 1 summarize the study design and show how each dataset was used.

GSE10935 contained paired papilloma and adjacent autologous laryngeal mucosa samples from 12 patients, profiled using Affymetrix microarrays [4]. GSE261589 contained single-cell RNA-sequencing data from papillomas of four patients with RRP and pharyngopalatine mucosa from three control donors [11]. GSE275788 contained paired papilloma samples collected before and after INO-3107 treatment from 17 participants in the single-arm phase 1/2 study; 16 post-treatment samples were collected at week 52 and one at week 47 [18]. The public files did not include a usable peripheral-blood RNA-expression dataset or verified information on individual clinical response.

In P1, papilloma tissue was compared with matched autologous laryngeal mucosa from the same patients. In P2, gene expression was compared between RRP and control donors within cell groups that contained enough data for analysis. S2 compared the proportions of the main recovered cell types between RRP and control donors. In P3, papilloma tissue collected after treatment was compared with pretreatment tissue from the same patients. In all analyses, the independent unit was the patient or donor, not the individual cell.

### 2.2. Analysis of Immune Gene Programs

We analyzed 12 predefined immune programs covering cyclic GMP-AMP synthase (cGAS)-stimulator of interferon genes (STING)/type I interferon (IFN-I) sensing, major histocompatibility complex (MHC) class I and II antigen presentation, CD8-associated and natural killer (NK)-associated cytotoxic programs, T-cell dysfunction/checkpoint activity, regulatory T-cell (Treg) responses, T helper 1/interferon gamma (Th1/IFN-γ), T helper 2 (Th2)-skewed responses, myeloid immunosuppressive/M2-like program, C-X-C motif chemokine receptor 3 (CXCR3)-associated immune-cell trafficking, and TGF-β immunosuppression. Four additional programs represented dendritic-cell activation, interleukin-10/signal transducer and activator of transcription 3 (IL-10/STAT3) suppression, basal epithelial/proliferative activity, and squamous differentiation (Supplementary Table S1).

A program was analyzed when at least five of its predefined genes were present in the dataset. Program scores were calculated with singscore, a single-sample method that ranks measured genes according to their expression within each sample [21]. With the centered scoring option, scores are expressed around zero. A higher centered score means that genes belonging to a given program tend to be more highly expressed relative to the other measured genes in that sample.

These scores summarize patterns of gene expression. They should not be interpreted as direct measurements of pathway activation, cell identity, or cellular function. To account for multiple testing, Benjamini–Hochberg false discovery rate (FDR) correction was applied separately to the primary and additional program sets [22].

### 2.3. Analysis of Paired Tissue Samples

For P1, the GPL571 and GPL96 microarray datasets were processed separately using robust multi-array averaging and mapped to gene symbols approved by the HUGO Gene Nomenclature Committee (HGNC) [23]. A total of 13,039 genes were measured on both platforms. Differences in immune program scores between papilloma and matched mucosa were tested with limma, a statistical framework for gene-expression analysis [24]. The main analysis included all 12 patient pairs. We also repeated the analysis using the 10 GPL571 pairs and a stricter nine-pair quality control subset to assess the robustness of the findings.

For P3, the public RNA-sequencing count data were mapped to HGNC gene symbols, filtered with edgeR, normalized using the trimmed mean of M values (TMM), and transformed with voom, which prepares RNA-sequencing count data for linear model analysis [25,26,27], before statistical testing. The analysis compared pretreatment and post-treatment papilloma tissue from the same patients. All 17 pairs were included in the main analysis. We also repeated the analysis in the predefined 14-pair quality control subset, in the 16 patients with post-treatment samples collected at week 52, and after omitting one patient pair at a time.

Because the source study did not include a control group, changes observed after INO-3107 cannot be interpreted as proof of a causal treatment effect. As additional analyses, we used mroast, a rotation-based test for predefined gene sets [28], and performed genome-wide differential gene-expression analysis.

### 2.4. Single-Cell Analysis and Recovered-Cell Composition

The seven single-cell datasets underwent quality control, including removal of likely doublets with scDblFinder [29]. After filtering, 50,754 cells remained for analysis. Cells were normalized, clustered, visualized with uniform manifold approximation and projection (UMAP), and assigned to nine broad cell groups using established cell markers, cluster-specific markers, the position of the clusters, and their distribution across donors [30]. These broad annotations were used for analysis but should not be interpreted as proof that a given transcript or function is unique to one cell type.

We next examined three broader cell groups: T/NK cells, myeloid cells, and epithelial cells. For each donor, gene counts from cells belonging to the same group were combined so that the donor, rather than each individual cell, was used as the unit of analysis. This approach is commonly referred to as pseudobulk analysis.

The T/NK and myeloid groups contained enough cells and sequencing data for comparison in all four RRP donors and all three controls. The epithelial group did not: only one control donor had sufficient data, so no comparison between RRP and control epithelial cells was performed.

For the T/NK and myeloid groups, counts were filtered, normalized using the trimmed mean of M values (TMM), and compared between RRP and control donors with limma. Because the dataset included only four RRP donors and three controls, we also used exact permutation testing based on all 35 possible ways of assigning the seven donors to the two groups.

The P2 analysis was planned as exploratory. A result was selected for further exploratory consideration when its effect was in the same direction as in P1 and also had either a nominal *p* value below 0.05 or a large effect size (|Cohen d| ≥ 0.8). This was used only to identify findings worth following up and was not treated as evidence of statistical significance. FDR correction remained the main method used to account for multiple testing.

For the recovered-cell composition analysis (S2), the proportion of each broad cell group was calculated separately for each donor. Differences between RRP and control donors were tested with exact permutation tests, with FDR correction across the nine cell groups.

Because there were only seven donors, the statistical resolution of this comparison was limited. Only 35 different group assignments were possible, which means that the smallest attainable two-sided exact *p* value was 0.0286. With nine cell groups being tested, an isolated result at this minimum would still not remain significant after FDR correction. We therefore interpret the absence of FDR-significant S2 results cautiously and do not treat it as evidence that no biological difference exists.

As an additional check, we repeated the composition analysis using a centered log-ratio transformation [31]. The reported cell proportions reflect the cells that were recovered after tissue dissociation, viability selection, capture, quality control, and annotation. They should not be interpreted as direct measurements of how many cells of each type were present in the original tissue.

### 2.5. Comparison Across Datasets

The three analyses used different types of comparisons. In P1, papilloma was compared with matched normal mucosa, and in P2, RRP samples were compared with control samples. In P3, tissue collected after INO-3107 treatment was compared with pretreatment tissue from the same patients.

When comparing P1 and P3, we considered the direction of change only. For example, a program score that was lower in untreated papilloma in P1 and higher after treatment in P3 was described as changing in the opposite direction. We did not perform a statistical test directly comparing the P1 and P3 effect estimates.

The same predefined gene programs were used across the datasets, but the measurements were not directly equivalent. P1 was based on whole-tissue expression, whereas P2 was based on gene expression within selected cell groups. For this reason, the absolute singscore values were not compared between datasets. We compared only the direction and overall pattern of the results. Effect estimates and *p* values were never combined across datasets. The rules used for these comparisons are summarized in Supplementary Table S6.

Throughout the manuscript, we therefore distinguish between findings that were statistically supported in the paired tissue datasets and observations from the smaller single-cell dataset, which are presented as exploratory.

For P1 and P3, effect size was expressed as paired d_z, calculated from the differences within each patient pair. Cohen d was used for P2, and Hedges g, which corrects for small sample size, was used for the recovered-cell composition analysis [32]. All tests were two-sided.

Age, sex, HPV genotype, disease severity, previous treatment, and clinical response were not included in the statistical models because these data were not consistently available or verified across the public datasets. Additional details on data processing, quality control, permutation testing, sensitivity analyses, and software are provided in the Supplementary Materials.

### 2.6. Reproducibility and Software

All analyses were performed in R 4.6.1 with Bioconductor 3.23. Package versions were recorded with renv so that the analysis environment could be reproduced.

The public datasets are available from the Gene Expression Omnibus (GEO), maintained by the National Center for Biotechnology Information (NCBI), under accession numbers GSE10935, GSE261589, and GSE275788 [33]. The analysis scripts, predefined analysis decisions, software files, checksums, and selected results are available in Zenodo (doi:10.5281/zenodo.22108809).

### 2.7. Use of Generative AI Tools

ChatGPT (OpenAI; GPT-5.6 Sol, https://developers.openai.com/api/docs/models/gpt-5.6-sol, accessed on 19 September 2026) was used to assist with drafting and debugging analysis code and with preparation of figure-generating code from verified result tables.

## 3. Results

### 3.1. Study Samples

Throughout the Results, P1 refers to the untreated paired-tissue analysis, P2 to the single-cell gene-expression analysis, S2 to the recovered-cell composition analysis, and P3 to the paired pre- and post-treatment analysis.

P1 included 24 microarrays from 12 patients, with paired papilloma and autologous laryngeal mucosa samples from each patient. A total of 13,039 genes were measured on both microarray platforms.

The single-cell dataset used for P2 and S2 included 50,754 cells from four RRP donors and three controls after quality control. These cells formed 29 clusters and were assigned to nine broad cell groups. Comparisons between RRP and control donors could be performed for the T/NK and myeloid groups, but not for epithelial cells because too few control samples contained sufficient epithelial data.

P3 included 34 tissue samples from 17 patients, with one pretreatment and one post-treatment sample from each patient. A total of 18,929 genes passed the expression filters.

All planned sensitivity analyses were completed, including the platform-specific analysis in P1, the predefined quality control subsets, the week-52 analysis in P3, and analyses repeated after omitting one patient or donor at a time.

### 3.2. Untreated Papillomas Show Lower Cytotoxic Program Scores and a Higher TGF-β Program Score

**Figure 2:**
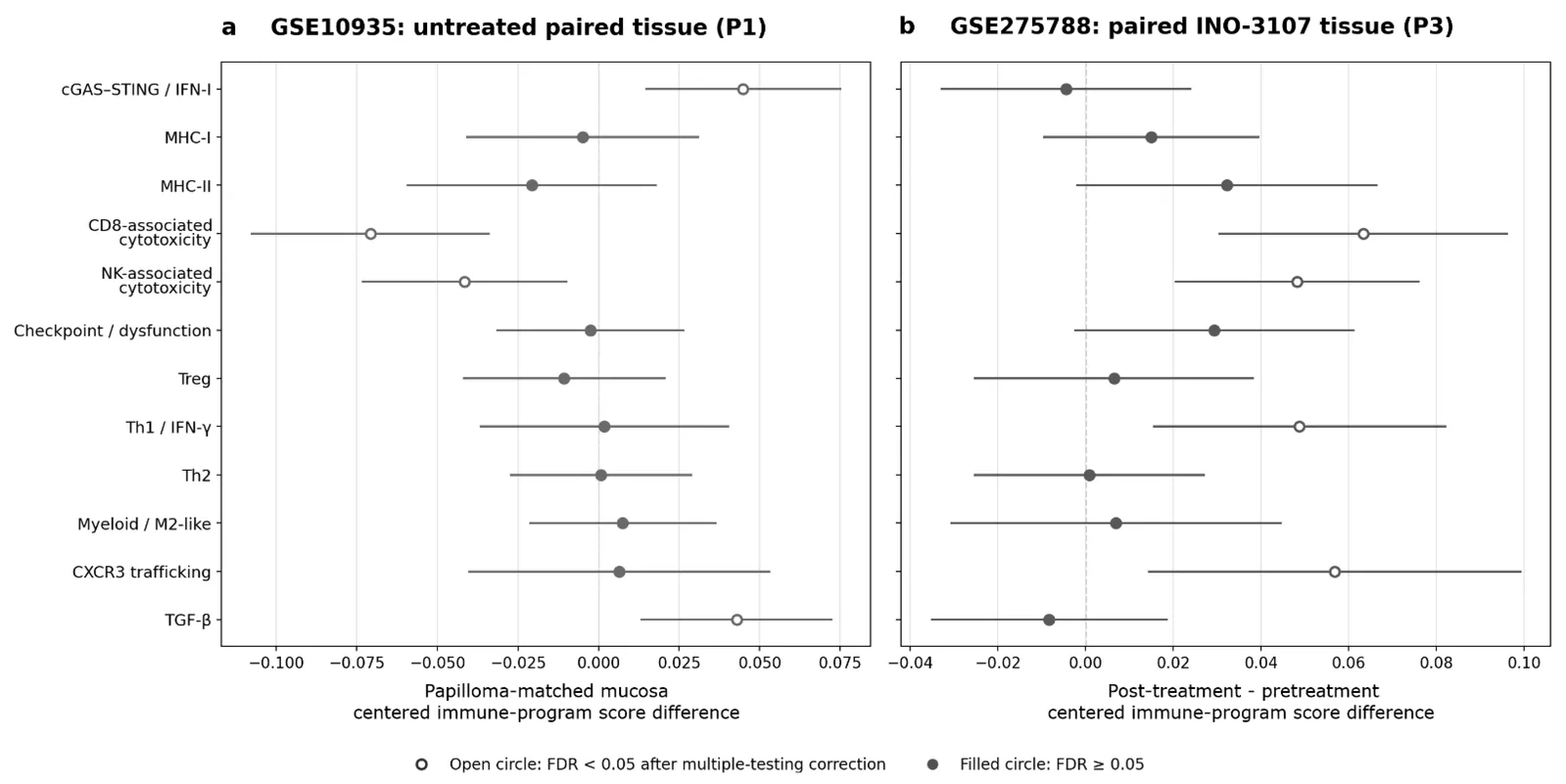
Immune program scores in untreated paired tissue and paired INO-3107 tissue datasets. (**a**) Papilloma-minus-matched-mucosa differences in GSE10935. (**b**) Post-treatment-minus-pretreatment differences in GSE275788 after INO-3107. Points show between-condition differences in centered singscores and bars show 95% confidence intervals. Positive values indicate higher centered program scores in papilloma (**a**) or after treatment (**b**), and negative values indicate lower scores. Open circles denote FDR < 0.05 after within-dataset Benjamini–Hochberg multiple-testing correction; filled circles denote FDR ≥ 0.05. The scores summarize transcriptional programs and are not direct functional measurements.

Four of the 12 primary immune programs differed between papilloma and matched autologous mucosa in P1 (Figure 2a; Supplementary Table S2).

The CD8-associated cytotoxic program score was lower in papilloma (difference = −0.071, 95% confidence interval (CI) −0.108 to −0.034; FDR = 0.0078; d_z = −1.02). The NK-associated cytotoxic program score was also lower (difference = −0.042, 95% CI −0.073 to −0.010; FDR = 0.0386; d_z = −0.81). Both differences remained in the same direction when the analysis was repeated after omitting one patient pair at a time. The CD8 result also remained significant when the analysis was restricted to the GPL571 platform. For the NK program, the difference remained lower in papilloma in the stricter nine-pair subset, although the effect was smaller.

The TGF-β immunosuppression program score was higher in papilloma (difference = 0.043, 95% CI 0.013 to 0.073; FDR = 0.0267; d_z = 0.99). The cGAS-STING/IFN-I program score was also higher (difference = 0.045, 95% CI 0.014 to 0.075; FDR = 0.0267; d_z = 0.98), which was opposite to our predefined expectation of weaker antiviral sensing. We therefore treat this as an unexpected finding in this dataset and do not interpret it as evidence of increased antiviral pathway activity.

The genome-wide paired analysis identified 890 genes with FDR < 0.05.

### 3.3. Exploratory Findings from the Single-Cell Dataset

**Figure 3:**
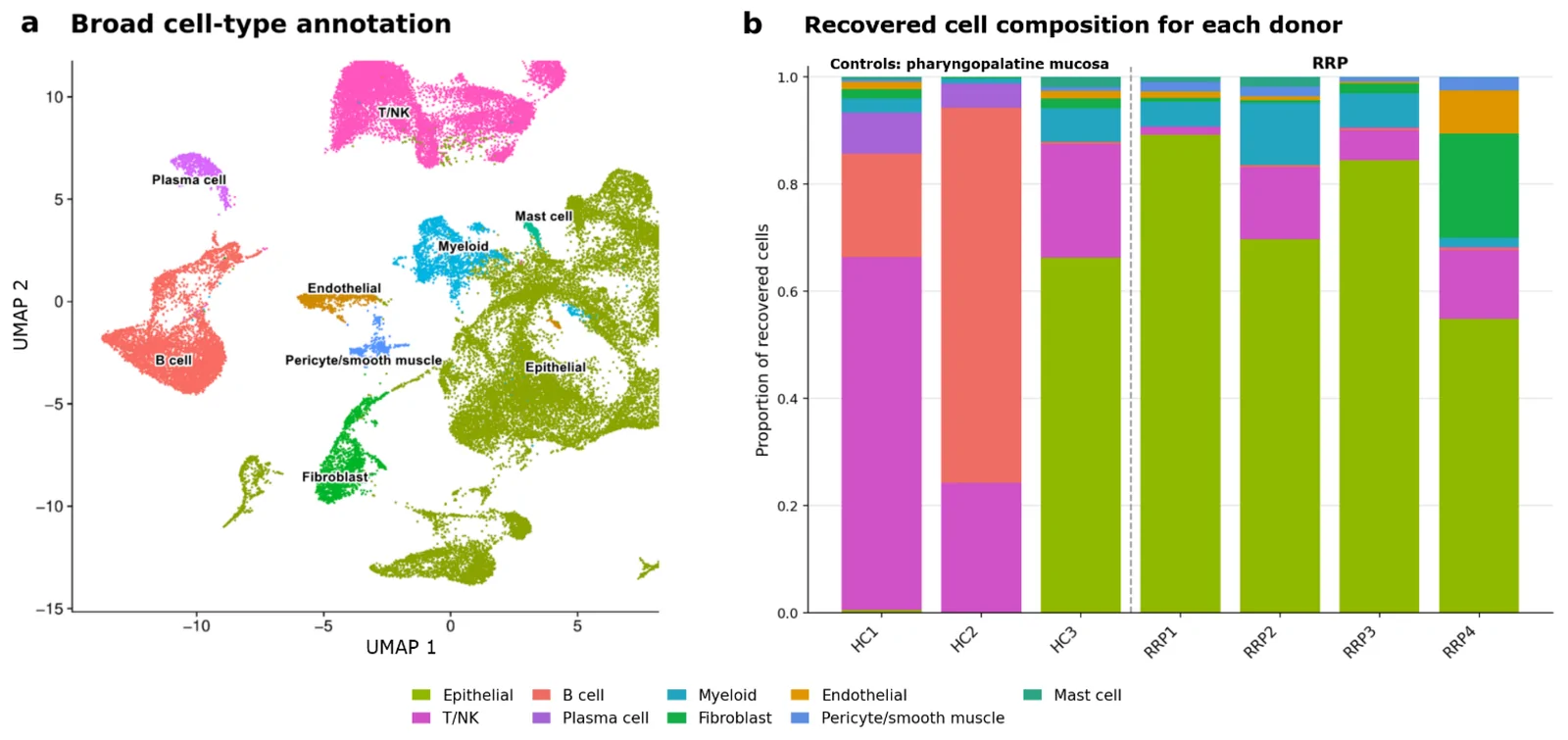
Single-cell atlas and donor-level recovered-cell composition of GSE261589. (**a**) UMAP of 50,754 quality-controlled cells after broad cell-class annotation. (**b**) Broad recovered-cell composition for each donor after dissociation, capture, quality control, and annotation. HC1-HC3 are control pharyngopalatine mucosa samples; RRP1-RRP4 are papilloma samples. The dashed line separates the two groups. The marked variation among control donors is visible in panel (**b**), and the displayed proportions are recovery measures rather than direct histological estimates of cellular abundance.

None of the broad recovered cell groups remained significant after FDR correction. The mean proportion of recovered T/NK cells was lower in RRP samples than in controls (8.35% vs. 37.08%; difference = −28.74 percentage points; nominal exact *p* = 0.0286; FDR = 0.1286; Hedges g = −1.47; Figure 3b; Supplementary Table S5). The centered log-ratio sensitivity analysis showed the same direction.

The control samples were highly heterogeneous. HC1 and HC2 contained only 0.56% and 0.03% recovered epithelial cells and were dominated by T/NK, B-cell, and plasma-cell populations, whereas HC3 contained 66.34% recovered epithelial cells. The recovered T/NK-cell ranges also did not overlap between groups, with 21.18–65.84% in controls and 1.56–13.42% in RRP.

These differences should be interpreted cautiously. The control tissue came from pharyngopalatine rather than matched laryngeal mucosa, the number of donors was small, and the proportion of recovered cells can be influenced by tissue dissociation, cell viability, capture, and annotation. The marked variation between control donors adds further uncertainty. We therefore treat the lower recovered T/NK-cell proportion as an exploratory observation rather than evidence of true histological depletion in RRP tissue.

**Figure 4:**
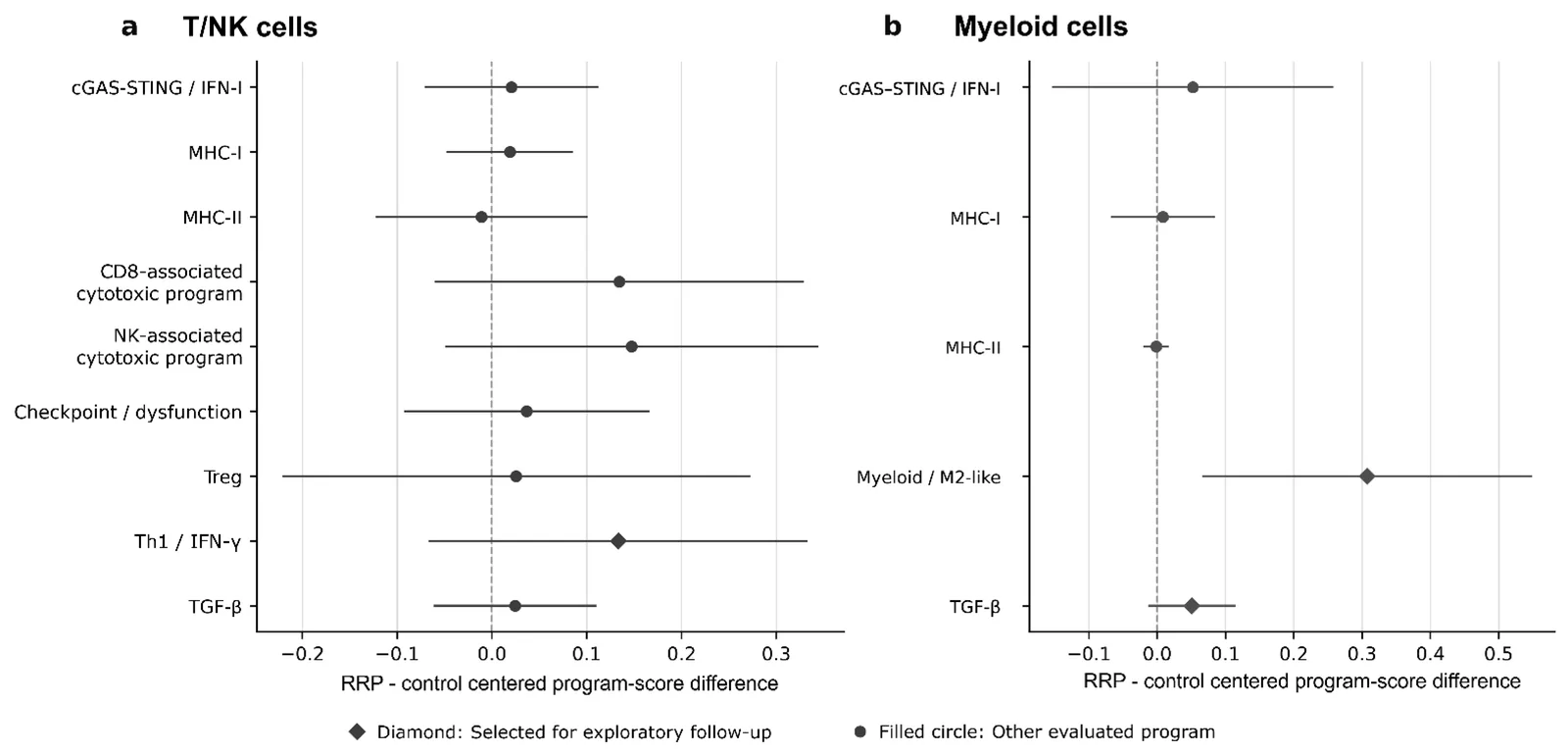
Exploratory comparison of program scores between RRP and control donors in GSE261589. (**a**) T/NK-cell analysis; (**b**) myeloid-cell analysis. Points show RRP-minus-control centered singscore differences and bars show 95% confidence intervals. Diamonds identify results selected for exploratory follow-up (direction aligned with the untreated P1 result plus nominal *p* < 0.05 or |Cohen d| ≥ 0.8); filled circles indicate other evaluable programs. The diamond is a hypothesis-generating marker, not a significance symbol. No P2 program reached FDR < 0.05.

No P2 immune program remained significant after FDR correction (Figure 4a; Supplementary Table S3). Within the broad T/NK-cell group, the CD8-associated and NK-associated cytotoxic program scores were higher in RRP than in controls (Cohen d = 1.10 and 1.19). This was the opposite direction to the lower whole-tissue scores seen in P1. The same pattern was seen after excluding RRP1, which had lower sequencing depth, and after repeating the analysis with one donor omitted at a time.

The Th1/IFN-γ program also showed a large difference in the same direction (difference = 0.133, 95% CI −0.065 to 0.332; *p* = 0.157; FDR = 0.471; d = 1.06). However, none of these comparisons remained significant after correction for multiple testing. The single-cell data therefore do not show that cytotoxic function is preserved, nor do they explain why the CD8- and NK-associated program scores were lower in whole papilloma tissue.

In the myeloid-cell analysis (Figure 4b), the M2-like program showed the largest difference between RRP and control donors (difference = 0.308, 95% CI 0.068 to 0.547; nominal *p* = 0.020; FDR = 0.102; exact *p* = 0.0286; d = 2.24). The TGF-β program was also higher in RRP (difference = 0.051, 95% CI −0.011 to 0.113; *p* = 0.093; FDR = 0.231; d = 1.46), in the same direction as in P1. The proportion of recovered myeloid cells was similar between groups (6.10% vs. 3.27%; *p* = 0.400).

Because no myeloid program or individual gene remained significant after FDR correction, these findings should be considered exploratory. They suggest patterns that may be worth testing in larger, anatomically matched cohorts, but they do not identify which cells produce the signal or what functional effect it has.

### 3.4. INO-3107 Is Associated with Increases in Cytotoxic and Trafficking Program Scores

GSE275788 comes from the phase 1/2 study of INO-3107, which reported fewer surgical procedures together with expansion and tissue trafficking of HPV-specific T cells [18].

In our analysis of paired samples collected before and after treatment, four immune program scores increased significantly (Figure 2b; Supplementary Table S4). These were the CD8-associated cytotoxic program (difference = 0.063, 95% CI 0.030 to 0.096; FDR = 0.0061; d_z = 0.90), the NK-associated cytotoxic program (difference = 0.048, 95% CI 0.020 to 0.076; FDR = 0.0087; d_z = 0.93), the Th1/IFN-γ program (difference = 0.049, 95% CI 0.015 to 0.082; FDR = 0.0231; d_z = 0.68), and the CXCR3-associated trafficking program (difference = 0.057, 95% CI 0.014 to 0.099; FDR = 0.0322; d_z = 0.57).

The same four program scores also increased significantly when the analysis was limited to the 16 patients with samples collected at week 52. The direction of these changes also remained the same when the analysis was repeated after omitting one patient pair at a time.

In the stricter 14-pair quality control subset, all four programs still showed higher scores after treatment, although the results did not remain significant after FDR correction. The additional mroast analysis showed the same overall pattern. No individual gene remained significant after FDR correction, suggesting that the observed changes involved groups of genes rather than a single dominant transcript.

The TGF-β program did not show a clear change after treatment (difference = −0.008, 95% CI −0.035 to 0.019; FDR = 0.803). The M2-like program also showed no statistically supported change.

Because the public dataset did not include verified information on individual clinical response and the original study did not include a control group, these data cannot show whether the observed immune changes were linked to clinical response or were caused by treatment itself.

### 3.5. Comparison of Findings Across Datasets

**Figure 5:**
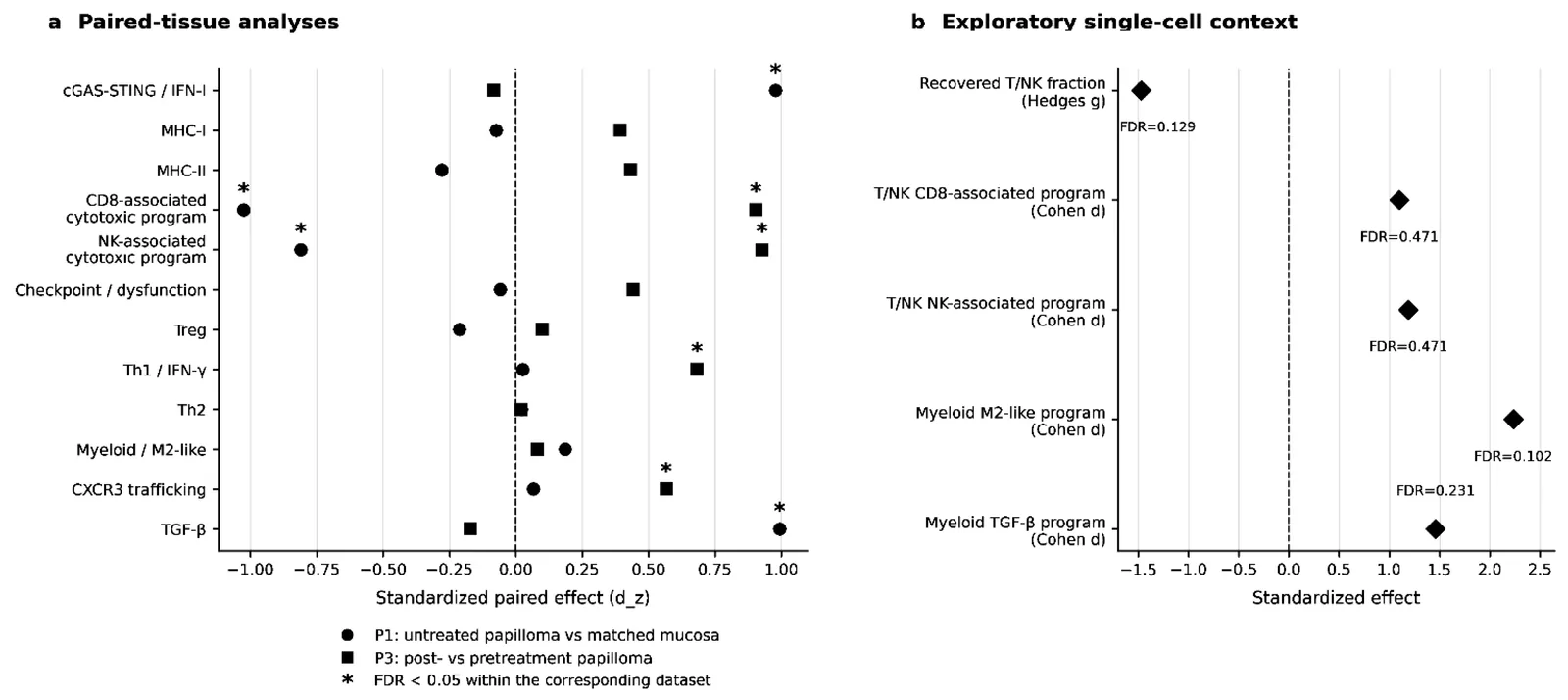
Separation of paired-tissue evidence from exploratory single-cell context. (**a**) Standardized paired effects (d_z) for the 12 primary immune programs in untreated papilloma versus matched autologous mucosa (P1; circles) and post- versus pretreatment papilloma after INO-3107 (P3; squares). Asterisks denote FDR < 0.05 within the corresponding paired dataset; effects were not pooled across datasets. (**b**) Selected standardized exploratory single-cell effects from GSE261589. The recovered T/NK fraction is shown as Hedges g, whereas within-compartment T/NK and myeloid program effects are shown as Cohen d and labeled accordingly in the panel. No P2/S2 result reached FDR < 0.05. The single-cell data provide hypothesis-generating context and do not establish the mechanism of the paired tissue differences.

The CD8-associated and NK-associated cytotoxic program scores were lower in untreated papilloma tissue in P1 and higher after INO-3107 in P3. Both findings were statistically significant within their respective datasets. We therefore describe the changes as occurring in opposite directions, while emphasizing that P1 and P3 were not compared directly in a statistical test (Figure 5; Table 2).

The single-cell analyses did not provide significant evidence after FDR correction to explain why the cytotoxic program scores were lower in whole papilloma tissue. The recovered-cell composition and the gene-expression patterns within individual cell groups are therefore used only as exploratory observations.

The TGF-β program score was higher in untreated papilloma tissue, but it did not show a clear change after INO-3107. We therefore cannot determine from these data whether this program changes after treatment.

The Th1/IFN-γ- and CXCR3-associated trafficking programs increased after treatment, although neither was clearly reduced in untreated papilloma tissue.

Taken together, the three datasets show a consistent pattern of differences in several immune programs, while the single-cell data remain insufficient to explain the underlying cellular mechanism.

## 4. Discussion

The main finding of this study is that different components of the local immune response in RRP do not follow the same pattern in untreated disease and after treatment. The three datasets address different parts of this question and provide different levels of evidence.

The GSE10935 analysis reproduced the pattern previously reported by DeVoti et al. [4]: CD8-associated and NK-associated cytotoxic program scores were lower in papilloma, whereas the TGF-β program score was higher. Because we analyzed the same patient cohort, this should be viewed as a reanalysis of the earlier finding rather than as independent confirmation.

The single-cell dataset adds exploratory information about possible cellular contributors to these whole-tissue patterns. The lower CD8- and NK-associated cytotoxic signal seen in whole tissue was not reproduced within the recovered T/NK-cell population. However, none of the single-cell comparisons remained significant after FDR correction. The small number of donors, the use of control tissue from a different anatomical site, and the marked variation between control samples further limit the conclusions that can be drawn from these data.

In the paired samples collected before and after INO-3107, CD8-associated cytotoxic, NK-associated cytotoxic, Th1/IFN-γ, and CXCR3-associated trafficking program scores all increased significantly after treatment, whereas TGF-β and M2-like suppressive programs showed no comparable statistically supported change. Immune activation after INO-3107 had already been reported in the original clinical study, so this is not a new finding by itself.

The contribution of the present study is the pattern that emerges when these three datasets are examined using the same predefined immune programs while each dataset is analyzed separately. Effector- and trafficking-related programs show the clearest treatment-associated changes, whereas suppressive programs change much less. At the same time, the reduced cytotoxic signal seen in untreated whole tissue is not reproduced within the recovered T/NK-cell population. Together, these findings suggest that immune remodeling in RRP is selective rather than uniform.

### 4.1. Cytotoxic Gene Programs and T/NK-Cell Findings

The lower CD8-associated and NK-associated program scores in P1 reproduce the pattern previously reported in the same GSE10935 cohort by DeVoti et al. [4] and are consistent with later studies describing impaired local antiviral immunity in RRP [5,10,12]. Because this is the same patient cohort, these findings should not be considered independent confirmation.

The single-cell dataset adds exploratory information about what may contribute to the whole-tissue pattern. Within the recovered T/NK-cell group, the CD8-associated and NK-associated program scores were not lower in RRP. However, none of the P2 comparisons remained significant after FDR correction, and the lower proportion of recovered T/NK cells in S2 was also not significant after correction for multiple testing.

We therefore do not conclude that fewer T/NK cells explain the lower whole-tissue cytotoxic program scores. Instead, the single-cell results raise the possibility that cell abundance, the mixture of T/NK-cell subsets, or their location within the tissue may contribute to the observed pattern. These possibilities require testing in an independent cohort.

The recovered-cell composition also shows why anatomically matched control tissue is important. The control samples came from pharyngopalatine rather than laryngeal mucosa and differed markedly from one another. HC1 and HC2 contained less than 1% recovered epithelial cells and were dominated by lymphoid cells, whereas 66.34% of the cells recovered from HC3 were epithelial. Although the T/NK-cell ranges did not overlap between RRP and control samples, this difference did not remain significant after FDR correction and may also reflect differences in anatomical site, donor characteristics, tissue processing, or cell recovery.

It is also important to distinguish gene-expression patterns from cell function. The terms “CD8-associated cytotoxic” and “NK-associated cytotoxic” refer to predefined gene programs. They do not mean that the cells have been shown to be functionally cytotoxic. Genes and granule components associated with cytotoxicity can also be present in other activated immune cells, and their presence alone does not demonstrate effective target-cell killing.

Testing these possibilities will require matched laryngeal control tissue together with spatial or histological assessment and functional assays.

### 4.2. TGF-β, Myeloid Programs, and Antiviral Sensing

The TGF-β findings differed from the pattern seen for the cytotoxic programs. In P1, the TGF-β immunosuppression program score was higher in untreated papilloma than in matched mucosa. In the single-cell dataset, the myeloid TGF-β and M2-like programs also showed relatively large differences in the same direction. However, neither remained significant after FDR correction. These findings therefore suggest possible changes in myeloid-associated gene expression but do not provide evidence for a defined suppressive macrophage population.

The direction of these findings is consistent with earlier studies describing altered monocyte and Langerhans-cell function in RRP and suppression of NK-cell activity by papilloma-derived TGF-β [8,9]. However, this biological consistency does not replace the need for independent validation.

TGF-β is also unlikely to have a simple role in RRP. In a trial of combined PD-L1 and TGF-β blockade, inhibition of TGF-β did not improve HPV-specific immunity and reduced an antiproliferative signal in papilloma epithelium [34]. In our single-cell dataset, the epithelial cells could not be compared between RRP and controls because only one control donor contained sufficient epithelial data. We therefore cannot determine from these data whether the TGF-β signal reflects immune suppression, effects on epithelial growth, or both. Addressing this question will require measurements of TGFB1 and phosphorylated SMAD (pSMAD) signaling in specific cell and tissue compartments.

The higher cGAS-STING/IFN-I program score in P1 also shows why gene-expression scores should not be interpreted as direct measures of pathway activity. HPV11 E7 can inhibit STING, TANK-binding kinase 1 (TBK1), and interferon regulatory factor 3 (IRF3) phosphorylation in infected epithelial cells [11]. At the same time, whole-tissue gene expression also reflects immune and stromal cells, which may contribute to the overall signal. Because the epithelial cells could not be compared between groups in P2, the unexpected P1 finding cannot be assigned to a specific cell type and will require protein-level and spatial validation.

### 4.3. Treatment-Associated Immune Remodeling

In P3, the CD8-associated cytotoxic, NK-associated cytotoxic, Th1/IFN-γ, and CXCR3-associated trafficking program scores all increased after INO-3107. This is consistent with the original clinical study, which reported expansion and tissue trafficking of HPV6/11-reactive T cells [18], and with later follow-up showing a sustained reduction in surgical burden [19].

Additional immune profiling from the same clinical program associated treatment response with activated HPV-specific cluster of differentiation 4 (CD4) T cells, Th1 and effector-memory responses in the airway, and human leukocyte antigen (HLA) class II expression [20]. Because these findings come from the same treatment program, they provide biological context rather than independent confirmation.

Our contribution is to examine the untreated and post-treatment tissue datasets using the same predefined immune programs. This allows the overall direction of the findings to be compared while keeping the statistical analyses separate. The CD8-associated and NK-associated program scores were lower in untreated papilloma in P1 and higher after treatment in P3. However, the two datasets were not compared directly, so this pattern should not be described as normalization or as evidence that these immune changes caused the clinical benefit. Similarly, the TGF-β program did not show a clear change after treatment, and the present data cannot establish whether it is altered by INO-3107.

The treatment landscape of RRP is changing rapidly. PRGN-2012/Papzimeos is now approved for adults, and clinical benefit in the pivotal study was associated with HPV6/11-specific T-cell responses [16,17]. Retrospective studies also suggest that prophylactic HPV vaccination, when used therapeutically, may reduce the need for surgery in some patients with established RRP [15].

These findings make local and systemic immune responses increasingly relevant to the management of RRP. Our analysis does not identify a clinical biomarker, but it suggests that future studies should examine not only HPV-specific immune responses in blood, but also the extent to which these cells reach papilloma tissue and interact with the local myeloid and TGF-β environment.

### 4.4. Strengths, Limitations, and Next Steps

This study has several strengths. The three datasets provide complementary information, and two include paired tissue samples from the same patients. In the single-cell analysis, the donor rather than the individual cell was used as the independent unit. We also analyzed recovered-cell composition separately from gene expression within individual cell groups and kept the statistical analyses of the three datasets separate.

The main limitations arise from the size and design of the available public datasets. The single-cell cohort included only four RRP donors and three controls, and none of the P2 or S2 findings remained significant after FDR correction. The control tissue was also pharyngopalatine rather than matched laryngeal mucosa, and the control samples showed marked variation in recovered-cell composition. These factors limit how far the single-cell findings can be interpreted.

Clinical information was not consistently available across the datasets, including HPV type, disease severity, previous treatment, and treatment response. In addition, the INO-3107 study did not include a control group, which limits causal interpretation of the post-treatment changes.

Finally, the immune program scores describe patterns of gene expression and should not be interpreted as direct measurements of cell function. Cytotoxicity-associated transcripts are not unique to CD8 T cells or NK cells, and their presence does not demonstrate effective target-cell killing.

A useful next step would be a prospective study collecting papilloma tissue, matched normal laryngeal mucosa, and blood from the same patients before treatment and during follow-up. Spatial or histological methods could then show where the relevant immune cells are located, while measurements of myeloid markers and TGFB1/pSMAD signaling could help clarify the source of the suppressive signal. Linking these findings to surgical frequency, intersurgical interval, Derkay score, and standardized treatment-response measures would allow the hypotheses raised here to be tested directly.

## 5. Conclusions

Taken together, the three complementary datasets provide a coherent picture of local immune changes in HPV6/11-associated RRP. Untreated papilloma tissue showed lower CD8- and NK-associated cytotoxic program scores together with a stronger TGF-β-associated suppressive program. The lower cytotoxic signal seen in whole tissue was not reproduced within the recovered T/NK-cell population in the single-cell dataset. Although these single-cell findings remain exploratory, the whole-tissue and T/NK-cell analyses did not show the same directional pattern.

After INO-3107, CD8- and NK-associated cytotoxic program scores increased together with Th1/IFN-γ and CXCR3-associated trafficking programs, whereas no comparable statistically supported change was seen in the TGF-β or M2-like suppressive programs. This indicates that different components of the local immune response do not respond to treatment in the same way. The clearest treatment-associated changes involved effector- and trafficking-related immunity, whereas no comparable statistically supported change was detected in the suppressive programs measured here.

These findings support a model in which treatment can strengthen antiviral effector immunity without a parallel reduction in suppressive features of the local tissue environment. This pattern may contribute to understanding why responses to immunotherapy can be incomplete or heterogeneous and provides a rationale for future studies of biomarkers, treatment monitoring, and combination approaches targeting more than one component of the local immune response. In particular, assessing both treatment-responsive effector immunity and local suppressive signals may be more informative than evaluating either component alone.

The main contribution of this study is the integration of untreated tissue, single-cell data, and paired pre- and post-treatment samples within a common predefined immune framework. This approach shows that immune changes in RRP are not coordinated across all compartments or functional programs. In particular, treatment-associated enhancement of effector immunity can occur without a comparable reduction in local suppressive signals. This distinction provides a more biologically informative framework for future biomarker development, treatment monitoring, and combination therapeutic strategies.

## Figures and Tables

**Table 1:** Public datasets and analytical roles.

Dataset	Analysis	Samples Analyzed	Comparison	Platform	Main Analysis
GSE10935	P1, untreated tissue	12 matched pairs	Papilloma vs. autologous adjacent laryngeal mucosa from the same patients	Affymetrix microarray	Paired analysis of gene expression and immune program scores; sensitivity analyses repeated after omitting one pair at a time
GSE261589	P2/S2, exploratory single-cell analysis	4 RRP donors and 3 controls; 50,754 cells	RRP papilloma vs. control pharyngopalatine mucosa	10× single-cell RNA sequencing	Gene expression summarized for each donor and cell group; exact permutation testing; recovered-cell composition; analyses repeated after omitting one donor at a time
GSE275788	P3, pre/post-treatment tissue	17 matched pre/post-treatment tissue pairs	Papilloma before vs. after INO-3107 treatment in the same patients	Tissue RNA sequencing	Paired analysis of gene expression and immune program scores; sensitivity analyses using week-52 samples, the predefined quality control subset, and analyses repeated after omitting one pair at a time

**Table 2:** Cross-dataset interpretation of the principal immune programs.

Program	Untreated Papilloma (P1)	Single-Cell Findings (P2/S2)	After INO-3107 (P3)	Interpretation
CD8-associated cytotoxic program	Lower than matched mucosa: FDR = 0.0078; d_z = −1.02	No single-cell result remained significant after FDR correction. The T/NK program score was higher in RRP, while the recovered T/NK-cell proportion was lower (FDR = 0.1286).	Higher after treatment: FDR = 0.0061; d_z = 0.90	The program score was lower in untreated papilloma and higher after treatment. Both findings were significant in their respective datasets, but P1 and P3 were not compared directly.
NK-associated cytotoxic program	Lower than matched mucosa: FDR = 0.0386; d_z = −0.81	No single-cell result remained significant after FDR correction. The T/NK program score was higher in RRP, while the composition finding remained exploratory.	Higher after treatment: FDR = 0.0087; d_z = 0.93	The program score was lower in untreated papilloma and higher after treatment. The single-cell data do not explain the cellular basis of this difference.
TGF-β immunosuppression program	Higher than matched mucosa: FDR = 0.0267; d_z = 0.99	The myeloid TGF-β program was higher in RRP (d = 1.46; FDR = 0.231), as was the M2-like program (d = 2.24; FDR = 0.102); both findings were exploratory.	No clear change after treatment: estimate = −0.008; FDR = 0.803	The higher TGF-β program score in untreated papilloma was statistically supported, but its cellular source and whether it changes after treatment remain unclear.

## Data Availability

The datasets analyzed in this study are publicly available in the NCBI Gene Expression Omnibus (GEO) under accession numbers GSE10935, GSE261589, and GSE275788. Analysis scripts, predefined analysis decisions, selected results, software files, checksums, and information documenting the source of the public data are available in Zenodo at https://doi.org/10.5281/zenodo.22108809. Original GEO files are not redistributed.

## Supplementary Materials

The following supporting information can be downloaded at https://www.mdpi.com/article/10.3390/v18101080/s1. The Supplementary Materials include detailed methods, the predefined immune program definitions, complete results for the three datasets, cell composition for each individual donor, cross-dataset comparisons, quality control and sensitivity analyses, and a graphical summary. Supplementary Methods S1–S9. Prespecified analysis plan; preprocessing and quality control for GSE10935, GSE261589, and GSE275788; immune-signature and donor-level analyses; recovered-cell composition; sensitivity analyses; and the rules for descriptive comparisons across datasets. Table S1. Prespecified immune-program definitions and constituent genes. The table lists each program’s analysis level, score direction, prespecified hypothesis, minimum number of genes required for evaluation, and constituent genes. Current HGNC-approved gene symbols are shown; the historical symbols used in the archived analysis files refer to the same genes and do not change program membership or the reported scores. Table S2. Complete primary persistence results for GSE10935. Immune-program scores are compared between papilloma and matched autologous laryngeal mucosa in 12 pairs. Estimates represent mean within-patient papilloma-minus-mucosa differences. Confidence intervals, nominal *p* values, false-discovery rates (FDRs), paired standardized effect sizes (d_z_), and the numbers of pairs with higher or lower scores in papilloma are reported. Table S3. Complete compartment-specific results for GSE261589. Donor-level aggregated expression in the T/NK and myeloid compartments is compared between four RRP donors and three control donors. Estimates represent mean RRP-minus-control score differences. FDR correction was performed separately for the nine evaluable T/NK programs and five myeloid programs. Exact *p* values were obtained from all 35 possible group-label allocations. The prespecified exploratory criterion required directional agreement with GSE10935 and either a nominal p value below 0.05 or an absolute Cohen d of at least 0.8; meeting this criterion was not treated as FDR-adjusted statistical significance. Table S4. Complete primary treatment-associated results for GSE275788. Immune-program scores are compared before and after treatment in 17 matched pairs. Estimates represent mean post-treatment-minus-pretreatment differences. Confidence intervals, nominal *p* values, FDRs, paired standardized effect sizes (d_z_), and observed directions are reported. Because the parent study was single-arm, the differences are described as treatment-associated changes. Table S5. Donor-level recovered-cell composition in GSE261589 and group-level sensitivity analysis. Part A gives the percentage of quality-controlled cells assigned to each broad cell class for every donor. Part B compares mean donor-level recovered-cell proportions between the RRP and control groups and presents a centered log-ratio sensitivity analysis. Exact two-sided *p* values were obtained from all 35 possible group-label allocations, with FDR correction across nine cell classes. These proportions describe cells recovered after dissociation, capture, quality control, and annotation; they are not direct estimates of histological cell abundance. Table S6. Comparison of immune-program findings across datasets. Results from the matched untreated-tissue comparison (P1, GSE10935), exploratory donor-level single-cell analysis (P2, GSE261589), and paired treatment-associated analysis (P3, GSE275788) are displayed together. Directional relationships are descriptive: effect estimates and *p* values were not pooled, and no statistical test directly compared effects across datasets. The P2 exploratory signals are distinguished from FDR-supported findings. Table S7. Prespecified analytical populations and sensitivity analyses. The table identifies the samples or donors included in each primary analysis and the planned restrictions used to examine the stability of findings, including platform and quality-control subsets, exclusion of a lower-depth T/NK donor library, and restriction to week-52 treatment samples. Figure S1. Graphical summary of the main findings. The paired untreated-tissue and post-treatment analyses are shown separately from the exploratory single-cell findings. In P1, CD8-associated and NK-associated cytotoxic program scores were lower, whereas the TGF-β immunosuppression program score was higher, in papilloma than in matched autologous laryngeal mucosa. These findings come from the previously studied GSE10935 cohort and do not constitute independent confirmation. No P2 or recovered-cell composition comparison remained significant after FDR correction; these results provide exploratory cellular context. In P3, CD8-associated, NK-associated, Th1/IFN-γ, and CXCR3-associated trafficking program scores increased after INO-3107. The P3 data did not establish a treatment-associated change in the TGF-β program. The opposite directions of the CD8- and NK-associated programs in P1 and P3 represent a descriptive cross-dataset observation and were not tested directly.

## Abbreviations

RRP, recurrent respiratory papillomatosis; HPV, human papillomavirus; NCBI, National Center for Biotechnology Information; GEO, Gene Expression Omnibus; scRNA-seq, single-cell RNA sequencing; CD8, cluster of differentiation 8; NK, natural killer; T/NK cells, T cells/natural killer cells; TGF-β, transforming growth factor beta; IFN-γ, interferon gamma; Th1, T helper 1; PD-1, programmed cell death protein 1; PD-L1, programmed death-ligand 1; cGAS, cyclic GMP-AMP synthase; STING, stimulator of interferon genes; MHC, major histocompatibility complex; CXCR3, C-X-C motif chemokine receptor 3; UMAP, uniform manifold approximation and projection; TMM, trimmed mean of M values; FDR, false discovery rate; HGNC, HUGO Gene Nomenclature Committee; CD4, cluster of differentiation 4; HLA, human leukocyte antigen; TBK1, TANK-binding kinase 1; IRF3, interferon regulatory factor 3; pSMAD, phosphorylated SMAD; Th2, T helper 2; Treg, regulatory T cell; IL-10, interleukin-10; STAT3, signal transducer and activator of transcription 3; CI, confidence interval; IFN-I, type I interferon.
