# Supplementary material for: HPV6/11 Persistence and Treatment-Associated Immune Remodeling in Recurrent Respiratory Papillomatosis: A Cross-Cohort Transcriptomic Analysis
Source: Viruses. 2026 Sep 30;18(10):1080. doi: 10.3390/v18101080 (PMC13709832; doi:10.3390/v18101080)
Supplement: Supplementary file 1 [file viruses-18-01080-s001.zip › viruses-4540321-supplementary.pdf]

Supplementary Materials

HPV6/11 Persistence and Treatment-Associated Immune Remodeling in Recurrent Respiratory Papillomatosis: A Cross-Cohort Transcriptomic Analysis

Supplementary Methods

S1. Prespecified Analysis Plan

The study included four complementary analyses: immune patterns in untreated papilloma tissue (P1), exploratory gene-expression analysis within cell groups from the single-cell dataset (P2), recovered-cell composition from the same dataset (S2), and immune changes after treatment (P3). Before examining the results, we defined the immune programs to be tested, their expected directions, the minimum number of genes required for analysis, the quality-control criteria, the approach to multiple-testing correction, the planned sensitivity analyses, and the rules used to compare findings across datasets. All publicly available samples that met the predefined requirements were included. Clinical variables that could not be verified from the original metadata were not reconstructed or inferred.

S2. GSE10935 microarray preprocessing and quality control

Raw CEL files generated on the GPL571 and GPL96 platforms were processed separately using robust multi-array averaging, with background correction, quantile normalization, and log2 transformation. Technical quality was assessed using complementary measures, including raw-intensity distributions, relative log-expression summaries, mean sample-wise Spearman correlations excluding self-correlations, and principal-component analysis based on non-constant, highly variable probesets. A sample was considered for technical exclusion only when at least two independent quality metrics showed an absolute robust z score greater than 4. Separation on principal-component analysis alone was not considered sufficient grounds for exclusion. Probe identifiers were mapped to HUGO Gene Nomenclature Committee (HGNC) gene symbols using platform-specific annotation packages. When several probesets mapped to the same gene, the probeset with the greatest across-sample interquartile range was selected as the representative measurement. The final shared expression space comprised 13,039 genes. All 12 matched papilloma–mucosa pairs were included in the main analysis. Prespecified sensitivity analyses included the 10 pairs profiled on GPL571 and a stricter nine-pair subset excluding P01, P02, and P09 because of extreme raw-intensity characteristics.

S3. GSE10935 analysis of immune signatures

Centered singscores were calculated for each sample using the package's centered scoring option. Singscore ranks measured genes within a sample and quantifies whether genes in a predefined program tend to occupy higher expression ranks; centered directional scores are expressed around zero. For every participant, the score obtained in matched normal mucosa was subtracted from the papilloma

score, yielding a within-patient papilloma-minus-mucosa difference. Mean paired differences were estimated using an intercept-only limma empirical-Bayes linear model. For each immune signature, we report the estimated mean difference, 95% confidence interval, nominal p value, Benjamini–Hochberg false-discovery rate (FDR), and paired standardized effect size (d_z, the mean within-pair difference divided by the standard deviation of paired differences). The stability of the primary findings was examined by repeating the analysis after sequentially omitting each matched pair. As an orthogonal gene-set analysis, mroast, a rotation-based gene-set test implemented in limma, was applied with 9999 rotations. Genome-wide paired differential-expression analysis was performed with limma and FDR correction and was treated as a secondary analysis.

S4. GSE261589 single-cell preprocessing and broad cell-type annotation

The seven raw 10x Genomics matrices were processed separately, using features annotated as human Gene Expression. Cells with fewer than 100 detected features or no detectable counts were removed before sample-level doublet detection with scDblFinder. Subsequent quality-control thresholds were adapted to the distribution of each sample using the median and three median absolute deviations, while keeping fixed biological and technical boundaries of at least 300 detected genes, at least 500 unique molecular identifiers (UMIs), no more than 6000 detected genes, and no more than 20% mitochondrial reads. Only cells meeting these criteria and classified as singlets were included. A total of 50,754 cells remained after quality control. Counts were log-normalized, 3000 variable features were selected, and the first 30 principal components were used to construct the nearest-neighbor graph, perform clustering at a resolution of 0.5, and generate the uniform manifold approximation and projection (UMAP) representation. The resulting 29 clusters were assigned to nine prespecified broad cell classes. Annotation was based on canonical lineage markers, the most informative cluster markers, the position of clusters within the UMAP, and representation across individual donors. Annotation was completed without condition-level biological testing so that knowledge of case–control differences did not influence cell-class assignment.

S5. Preparation and Analysis of Donor-Level Single-Cell Data

Epithelial, T/NK, and myeloid compartments were initially considered for donor-level aggregated expression analysis (often termed pseudobulk analysis). Raw single-cell counts were summed within each donor and compartment, preserving the donor rather than the individual cell as the independent experimental unit. A donor-compartment sample was included when it contained at least 50 cells and at least 100,000 raw unique molecular identifiers (UMIs). A compartment was compared between groups only when at least three eligible RRP donors and three eligible control donors were available. Both the T/NK and myeloid compartments met these requirements and included all four RRP and all three control donors. The epithelial compartment was not analyzed further because only one control donor met the predefined requirements, making a meaningful group comparison impossible. Within each eligible compartment, raw counts were summed at the donor level. Ensembl features mapping to the same HGNC gene symbol were combined, lowly expressed genes were removed using edgeR::filterByExpr, and library-size differences were normalized with the trimmed mean of M-values (TMM) method. Log counts per million were calculated using a prior count of 2. After filtering, 11,101 genes remained in the T/NK compartment and 8395 in the myeloid compartment. Quality control included total library size, number of detected genes, TMM normalization factor, mean correlation with the remaining donor libraries, and distance in principal-component space. No donor

library met the predefined requirement for exclusion based on two independent quality metrics. The lower-depth T/NK library from donor RRP1 was therefore included in the main analysis and excluded only in the prespecified three-versus-three sensitivity analysis.

S6. P2 statistical analysis, permutation testing, and sensitivity analyses

Centered singscores were compared between RRP and control donors using limma to estimate the mean RRP-minus-control difference. Multiple-testing correction was performed separately within the two predefined analytical families: nine evaluable primary signatures in the T/NK compartment and five in the myeloid compartment.

Because of the small number of donors, P2 was regarded as an exploratory compartment-level analysis. The prespecified follow-up criterion required the direction of the P2 effect to agree with that observed in P1 and, in addition, either a nominal p value below 0.05 or an absolute Cohen d of at least 0.8. This rule identified large and directionally coherent signals but was not treated as statistical significance or as a substitute for FDR-adjusted evidence. Exact two-sided permutation tests were used to avoid reliance on large-sample approximations. For the main four-versus-three comparison, all 35 possible allocations of the seven donors were enumerated. In the T/NK sensitivity analysis excluding RRP1, all 20 possible three-versus-three allocations were evaluated. Robustness was further examined by repeating normalization and signature scoring after sequentially omitting each donor. Gene-level confirmation used voomWithQualityWeights followed by mroast with 9999 rotations. Genome-wide differential-expression analysis was considered secondary.

S7. Recovered-cell composition analysis

To provide a complementary view of the single-cell dataset, we examined the distribution of the nine broad cell classes across individual donors. For each donor, the number of cells assigned to a given class was divided by the total number of cells remaining after quality control. The primary quantity of interest was the difference between RRP and control groups in mean donor-level recovered-cell proportion. Exact two-sided p values were obtained by enumerating all 35 possible group-label allocations, and FDR correction was applied across the nine prespecified cell classes. Because proportions derived from single-cell recovery are compositional, a centered log-ratio sensitivity analysis was also performed. A pseudocount of 0.5 cell was added to each class before closure and centered log-ratio transformation, after which the same exact permutation procedure was applied. These analyses describe the relative composition of cells recovered by the single-cell experiment after dissociation, capture, quality control, and annotation. They were not interpreted as direct measurements of histological cell abundance in papilloma tissue.

S8. GSE275788 preprocessing and analysis of treatment-associated change

The publicly available long-format tissue count file was converted to a gene-by-sample expression matrix. Stable feature identifiers were mapped to unique HGNC gene symbols, and a paired model was specified with participant identity and post-treatment status. Lowly expressed genes were removed using edgeR::filterByExpr, leaving 18,929 genes for analysis. Libraries were then TMM-normalized and transformed using voom, which generates log-counts per million with precision weights for linear modeling. All 17 complete pre- and post-treatment pairs were included in the main analysis. Three

complementary sensitivity analyses were prespecified. The first used a 14-pair quality-control subset excluding Pt1, Pt17, and Pt26. The second restricted the analysis to samples collected at week 52 by excluding the single week-47 pair. The third repeated the analysis after sequentially omitting each of the 17 pairs. Centered singscores were analyzed using paired limma models. The primary gene sets were additionally examined with mroast using 9999 rotations, while genome-wide paired differential expression was evaluated using voom-limma as a secondary analysis. Because the parent clinical study was single-arm, all pre/post differences are described as treatment-associated changes. They were not interpreted as causal treatment effects or as evidence of complete immune normalization.

S9. Comparison Across Datasets, Effect Sizes, and Reproducibility

The three datasets were analyzed separately and were not combined statistically. P1 and P2 compared disease samples with reference samples, whereas P3 compared post-treatment with pretreatment tissue. P1 and P2 effects were described as directionally concordant when their signs agreed. A P3 post-treatment change was described as directionally opposite to the corresponding P1 disease-associated difference when the signs differed. These were descriptive sign comparisons only; no statistical test directly compared effect estimates across datasets. Table S6 was used to organize findings across the three datasets, and neither effect estimates nor p values were pooled. Paired standardized effect sizes (d_z) were used for P1 and P3, Cohen d for P2, and Hedges g, which corrects for small sample size, for recovered-cell composition. All statistical tests were two-sided. Analyses were performed in R 4.6.1 with Bioconductor 3.23. Package versions were recorded with renv to support reproducibility. The random-number seed was fixed at 20260727. Input inventories, file checksums, quality-control decisions, software versions, session information, and complete result tables were archived as part of the reproducibility record.

Supplementary Tables

Table S1. Prespecified immune-program definitions and constituent genes.

Signature	Immune program	Analysis level	Score direction	Prespecified hypothesis	Minimum genes	Genes (HGNC)
SIG01	cGAS-STING / type I interferon sensing	Primary	Higher = stronger antiviral sensing	Expected lower in RRP; higher after effective therapy	5	CGAS; STING1; TBK1; IRF3; IFNB1; RIGI; IFIH1; MAVS; STAT1; STAT2; IRF7; ISG15; IFIT1; IFIT2; IFIT3; MX1; OAS1; OAS2; OAS3; BST2
SIG02	Antigen presentation - MHC class I	Primary	Higher = stronger class I antigen presentation	Expected lower in RRP; higher after therapy	5	B2M; HLA-A; HLA-B; HLA-C; TAP1; TAP2; TAPBP; PSMB8; PSMB9; NLRC5
SIG03	Antigen presentation - MHC class II	Primary	Higher = stronger class II antigen presentation	Direction evaluated; local induction after therapy anticipated	5	HLA-DRA; HLA-DRB1; HLA-DPA1; HLA-DPB1; HLA-DQA1; HLA-DQB1; CD74; CIITA
SIG04	CD8-associated cytotoxic program	Primary	Higher = higher CD8-associated cytotoxic program score	Lower score expected in untreated tissue; higher after therapy	5	CD8A; CD8B; NKG7; GNLY; PRF1; GZMA; GZMB; GZMH; CTSW; CCL5
SIG05	NK-associated cytotoxic program	Primary	Higher = higher NK-associated cytotoxic program score	Lower score expected in RRP; higher after therapy	5	NKG7; GNLY; PRF1; GZMB; GZMH; KLRD1; KLRK1; NCR1; FCGR3A; TYROBP
SIG06	T-cell dysfunction / checkpoint program	Primary	Higher = stronger dysfunction/checkpoint state	Expected higher in RRP; direction after therapy may be biphasic and interpreted alongside cytotoxic-program scores	5	PDCD1; CTLA4; LAG3; TIGIT; HAVCR2; TOX; ENTPD1; CD244; BTLA; EOMES
SIG07	Regulatory T-cell program	Primary	Higher = stronger Treg state	Expected higher in RRP; lower relative to effector activation after therapy	5	FOXP3; IL2RA; CTLA4; IKZF2; TIGIT; CCR8; TNFRSF18; LRRC32
SIG08	Th1/IFN-γ program	Primary	Higher = stronger type 1 immunity	Expected lower in RRP; higher after therapy	5	TBX21; IFNG; CXCR3; STAT4; IL12RB2; CCL5; CXCL9; CXCL10
SIG09	Th2-skewed program	Primary	Higher = stronger type 2 state	Expected higher in persistent RRP; lower or balanced after therapy	5	GATA3; IL4; IL5; IL13; CCR4; PTGDR2; IL1RL1; STAT6
SIG10	Myeloid immunosuppressive / M2-like program	Primary	Higher = stronger suppressive myeloid state	Expected higher in RRP; lower after therapy	5	CD163; MRC1; MSR1; ARG1; IL10; TGFB1; CCL18; VSIG4; MARCO
SIG11	Dendritic-cell antigen-presenting activation	Secondary	Higher = stronger DC activation	Expected reduced in RRP or increased after therapy	5	FCER1A; CD1C; CLEC10A; HLA-DRA; CD74; IL12B; CCR7; CD80; CD86
SIG12	CXCR3-axis lymphocyte trafficking	Primary	Higher = stronger effector-cell recruitment	Expected lower in RRP; higher after therapy	5	CXCL9; CXCL10; CXCL11; CXCR3; CCL5; CCR5
SIG13	TGF-β immunosuppression	Primary	Higher = higher TGF-β immunosuppression program score	Expected higher in RRP; lower after therapy	5	TGFB1; TGFBR1; TGFBR2; SMAD2; SMAD3; SMAD4; SERPINE1; CCN2; PMEPA1
SIG14	IL-10 / STAT3 suppressive signalling	Secondary	Higher = stronger IL-10 suppression	Expected higher in RRP; lower after therapy	5	IL10; IL10RA; IL10RB; STAT3; SOCS3; BCL3; DUSP1
SIG15	Basal epithelial / proliferative program	Secondary	Higher = basal/proliferative epithelial state	Expected higher in papilloma; reduction after effective local response	5	KRT5; KRT14; KRT17; KRT19; TP63; MKI67; PCNA
SIG16	Squamous differentiation program	Secondary	Higher = terminal squamous differentiation	Direction data-driven but locked after preregistration	5	IVL; KRT1; KRT10; DSG1; FLG; LOR; SPRR1A; SPRR1B

Nomenclature note: current HGNC-approved symbols are shown. The archived analysis files used historical symbols for four unchanged gene identities: MB21D1→CGAS, TMEM173→STING1, DDX58→RIGI, and CTGF→CCN2. This nomenclature update does not change gene membership or any reported score.

Table S2. Complete primary persistence results for GSE10935.

Signature	Immune program	Estimate	95% CI, lower	95% CI, upper	p value	FDR	Paired d_z	Pairs higher	Pairs lower	P1 interpretation
SIG01	cGAS-STING / type I interferon sensi ng	0.04486	0.01445	0.07528	0.00566	0.02665	0.97826	10	2	Significant but opposite to prespecified polarity
SIG02	Antigen presentation – MHC class I	-0.00495	-0.04113	0.03124	0.77984	0.95623	-0.07492	5	7	Not statistically supported
SIG03	Antigen presentation – MHC class II	-0.02074	-0.05955	0.01808	0.28051	0.67323	-0.27912	4	8	Not statistically supported; direction-evaluated s ignature
SIG04	CD8-associated cytotoxic program	-0.07077	-0.1079	-0.03364	0.00065	0.00777	-1.0248	2	10	Statistically supported
SIG05	NK-associated cytotoxic program	-0.04154	-0.0734	-0.00968	0.01286	0.03859	-0.80959	2	10	Statistically supported
SIG06	T-cell dysfunction / checkpoint progra m	-0.00244	-0.0316	0.02672	0.86408	0.95623	-0.05984	5	7	Not statistically supported
SIG07	Regulatory T-cell program	-0.01064	-0.04215	0.02086	0.49176	0.95623	-0.21284	5	7	Not statistically supported
SIG08	Th1/IFN-γ program	0.00185	-0.03675	0.04046	0.92174	0.95623	0.02517	6	6	Not statistically supported
SIG09	Th2-skewed program	0.00076	-0.02749	0.02901	0.95623	0.95623	0.02058	5	7	Not statistically supported
SIG10	Myeloid immunosuppressive / M2-like program	0.0075	-0.0216	0.03659	0.59917	0.95623	0.18502	6	6	Not statistically supported
SIG12	CXCR3-axis lymphocyte trafficking	0.00649	-0.04045	0.05342	0.7775	0.95623	0.06617	6	6	Not statistically supported
SIG13	TGF-β immunosuppression	0.04286	0.01313	0.0726	0.00666	0.02665	0.99314	9	3	Statistically supported

Table S3. Complete compartment-specific P2 results.

Signature	Immune program	Compartment	RRP-control es timate	95% CI, low er	95% CI, upp er	p value	FDR	Cohen d	Exact p	Exploratory P2 criterion met	Criterion basis
SIG01	cGAS-STING / type I interferon sensi ng	T/NK	0.02074	-0.06964	0.11112	0.60526	0.77819	0.45871	0.68571	No	Direction concordant, but neither nomina l p<0.05 nor |Cohen d|>=0.8
SIG02	Antigen presentation – MHC class I	T/NK	0.01904	-0.04641	0.0845	0.51477	0.77819	1.04416	0.31429	No	Direction not concordant with P1
SIG03	Antigen presentation – MHC class II	T/NK	-0.01096	-0.12142	0.0995	0.82172	0.82172	-0.17733	0.91429	No	Direction concordant, but neither nomina l p<0.05 nor |Cohen d|>=0.8
SIG04	CD8-associated cytotoxic program	T/NK	0.13455	-0.05874	0.32785	0.14425	0.47139	1.10191	0.22857	No	Direction not concordant with P1
SIG05	NK-associated cytotoxic program	T/NK	0.14749	-0.04814	0.34311	0.11819	0.47139	1.19198	0.17143	No	Direction not concordant with P1
SIG06	T-cell dysfunction / checkpoint progra m	T/NK	0.03672	-0.0913	0.16474	0.52051	0.77819	0.4878	0.51429	No	Direction not concordant with P1
SIG07	Regulatory T-cell program	T/NK	0.02566	-0.21983	0.27115	0.81245	0.82172	0.16227	0.77143	No	Direction not concordant with P1

Signature	Immune program	Compartment	RRP-control estimate	95% CI, lower	95% CI, upper	p value	FDR	Cohen d	Exact p	Exploratory P2 criterion met	Criterion basis
SIG08	Th1/IFN-γ program	T/NK	0.13314	-0.06526	0.33153	0.15713	0.47139	1.0595	0.25714	Yes	Direction concordant with P1; |Cohen d|>=0.8
SIG13	TGF-β immunosuppression	T/NK	0.02455	-0.05989	0.10899	0.51499	0.77819	0.6164	0.48571	No	Direction concordant, but neither nominal p<0.05 nor |Cohen d|>=0.8
SIG01	cGAS-STING / type I interferon sensing	Myeloid	0.05206	-0.15217	0.2563	0.55375	0.85076	0.44511	0.6	No	Direction concordant, but neither nominal p<0.05 nor |Cohen d|>=0.8
SIG02	Antigen presentation – MHC class I	Myeloid	0.00851	-0.06606	0.08308	0.78839	0.85076	0.20268	0.74286	No	Direction not concordant with P1
SIG03	Antigen presentation – MHC class II	Myeloid	-0.00136	-0.01833	0.01562	0.85076	0.85076	-0.28344	0.68571	No	Direction concordant, but neither nominal p<0.05 nor |Cohen d|>=0.8
SIG10	Myeloid immunosuppressive / M2-like program	Myeloid	0.30751	0.06779	0.54722	0.0203	0.10151	2.2382	0.02857	Yes	Direction concordant with P1; nominal p<0.05 and |Cohen d|>=0.8
SIG13	TGF-β immunosuppression	Myeloid	0.05084	-0.01148	0.11317	0.09252	0.2313	1.4624	0.11429	Yes	Direction concordant with P1; |Cohen d|>=0.8

Table S4. Complete primary treatment-associated results for GSE275788.

Signature	Immune program	Estimate	95% CI, lower	95% CI, upper	p value	FDR	Paired d_z	Observed direction
SIG01	cGAS-STING / type I interferon sensing	-0.00447	-0.03307	0.02413	0.75113	0.81942	-0.0823	Lower after treatment
SIG02	Antigen presentation – MHC class I	0.01499	-0.00969	0.03967	0.22388	0.38379	0.39243	Higher after treatment
SIG03	Antigen presentation – MHC class II	0.03219	-0.00222	0.0666	0.06561	0.14029	0.43113	Higher after treatment
SIG04	CD8-associated cytotoxic program	0.06336	0.03032	0.0964	0.00051	0.00606	0.90401	Higher after treatment
SIG05	NK-associated cytotoxic program	0.04821	0.02023	0.07618	0.00145	0.00872	0.92739	Higher after treatment
SIG06	T-cell dysfunction / checkpoint program	0.02938	-0.00258	0.06134	0.07014	0.14029	0.44238	Higher after treatment
SIG07	Regulatory T-cell program	0.00652	-0.02542	0.03847	0.67902	0.81942	0.09829	Higher after treatment
SIG08	Th1/IFN-γ program	0.04878	0.01533	0.08224	0.00579	0.02315	0.68241	Higher after treatment
SIG09	Th2-skewed program	0.00086	-0.02549	0.02721	0.94724	0.94724	0.01889	Higher after treatment
SIG10	Myeloid immunosuppressive / M2-like program	0.00695	-0.03076	0.04467	0.70853	0.81942	0.08152	Higher after treatment
SIG12	CXCR3-axis lymphocyte trafficking	0.05678	0.01423	0.09933	0.01073	0.03218	0.56607	Higher after treatment
SIG13	TGF-β immunosuppression	-0.00829	-0.03533	0.01875	0.53518	0.80277	-0.17169	Lower after treatment

Table S5. Donor-level recovered-cell composition in GSE261589 and group-level raw-proportion/centered log-ratio sensitivity analysis.

Part A. Donor-level percentages of recovered broad cell classes.

Donor	Recovered cells, n	Epithelial	T/NK	B cell	Plasma cell	Myeloid	Fibroblast	Endothelial	Pericyte/smooth muscle	Mast cell
HC1	7,971	0.56	65.84	19.28	7.65	2.62	1.77	1.35	0.44	0.48

Donor	Recovered cells, n	Epithelial	T/NK	B cell	Plasma cell	Myeloid	Fibroblast	Endothelial	Pericyte/smooth muscle	Mast cell
HC2	6,761	0.03	24.23	70.00	4.51	0.99	0.16	0.03	0.01	0.03
HC3	8,351	66.34	21.18	0.36	0.05	6.20	1.83	1.48	0.67	1.88
RRP1	6,778	89.19	1.56	0.01	0.00	4.62	0.69	1.25	1.77	0.90
RRP2	5,695	69.76	13.42	0.35	0.02	11.62	0.53	0.70	1.84	1.76
RRP3	7,056	84.52	5.51	0.43	0.17	6.39	1.81	0.24	0.88	0.04
RRP4	8,142	54.86	12.90	0.32	0.22	1.78	19.34	8.12	2.39	0.06

Values are percentages of all recovered quality-controlled cells for each donor. HC1-HC3 are control pharyngopalatine mucosa samples; RRP1-RRP4 are papilloma samples. Percentages reflect recovery after dissociation, capture, quality control, and annotation and are not direct histological abundance estimates.

Part B. Group-level raw-proportion results and centered log-ratio sensitivity analysis.

Cell class	Mean RRP	Mean control	Difference, pp	Hedges g	Exact p	FDR	CLR difference	Hedges g (CLR)	Exact p (CLR)	FDR (CLR)
T/NK	0.08347	0.37083	-28.73591	-1.47283	0.02857	0.12857	-1.6619	-2.06561	0.02857	0.12857
B cell	0.00278	0.29882	-29.60441	-1.09279	0.05714	0.12857	-3.77981	-1.38813	0.08571	0.19286
Epithelial	0.74584	0.22311	52.27284	1.63062	0.05714	0.12857	4.16789	1.65521	0.05714	0.17143
Pericyte/smooth muscle	0.01722	0.00375	1.34714	2.13853	0.05714	0.12857	2.14154	2.47775	0.02857	0.12857
Plasma cell	0.00102	0.04071	-3.96843	-1.37959	0.08571	0.15429	-3.1618	-1.19304	0.14286	0.25714
Myeloid	0.06104	0.03272	2.83174	0.65694	0.4	0.6	0.6609	0.62925	0.4	0.51429
Fibroblast	0.05595	0.01255	4.34002	0.51111	0.77143	0.9	0.82741	0.63212	0.45714	0.51429
Endothelial	0.02579	0.00956	1.62249	0.46665	0.8	0.9	0.99687	0.67468	0.37143	0.51429
Mast cell	0.0069	0.00795	-0.10549	-0.10095	0.91429	0.91429	-0.1911	-0.09244	0.91429	0.91429

Table S6. Comparison of immune-program findings across datasets.

Signature	Immune program	P1 estimate	P1 FDR	P1 d_z	P2 cell group(s) analyzed	P2 exploratory signal	P3 estimate	P3 FDR	P3 d_z	Comparison across datasets	Overall interpretation
SIG01	cGAS-STING / type I interferon sensing	0.04486	0.02665	0.97826	T/NK;Myeloid		-0.00447	0.81942	-0.0823		Unexpected P1 finding; treatment pattern unclear
SIG02	Antigen presentation – MHC class I	-0.00495	0.95623	-0.07492	T/NK;Myeloid		0.01499	0.38379	0.39243		No consistent pattern across datasets
SIG03	Antigen presentation – MHC class II	-0.02074	0.67323	-0.27912	T/NK;Myeloid		0.03219	0.14029	0.43113		No consistent pattern across datasets
SIG04	CD8-associated cytotoxic program	-0.07077	0.00777	-1.0248	T/NK		0.06336	0.00606	0.90401	Lower in P1 and higher in P3; both findings were statistically significant within their respective datasets. P1 and P3 were not compared directly.	Supported in P1 and P3; P2 exploratory

Signature	Immune program	P1 estimate	P1 FDR	P1 d_z	P2 cell group(s) analyzed	P2 exploratory signal	P3 estimate	P3 FDR	P3 d_z	Comparison across datasets	Overall interpretation
SIG05	NK-associated cytotoxic program	-0.04154	0.03859	-0.80959	T/NK		0.04821	0.00872	0.92739	Lower in P1 and higher in P3; both findings were statistically significant within their respective datasets. P1 and P3 were not compared directly.	Supported in P1 and P3; P2 exploratory
SIG06	T-cell dysfunction / checkpoint program	-0.00244	0.95623	-0.05984	T/NK		0.02938	0.14029	0.44238		No consistent pattern across datasets
SIG07	Regulatory T-cell program	-0.01064	0.95623	-0.21284	T/NK		0.00652	0.81942	0.09829		No consistent pattern across datasets
SIG08	Th1/IFN-γ program	0.00185	0.95623	0.02517	T/NK	T/NK	0.04878	0.02315	0.68241		Increase after treatment supported in P3; P2 exploratory
SIG09	Th2-skewed program	0.00076	0.95623	0.02058			0.00086	0.94724	0.01889		No consistent pattern across datasets
SIG10	Myeloid immunosuppressive / M2-like program	0.0075	0.95623	0.18502	Myeloid	Myeloid	0.00695	0.81942	0.08152		Exploratory P2 finding only
SIG12	CXCR3-axis lymphocyte trafficking	0.00649	0.95623	0.06617			0.05678	0.03218	0.56607		Increase after treatment supported in P3
SIG13	TGF-β immunosuppression	0.04286	0.02665	0.99314	T/NK;Myeloid	Myeloid	-0.00829	0.80277	-0.17169	Higher in P1; exploratory myeloid signal in P2; P3 does not establish a treatment-associated TGF-β change.	Supported in P1; P2 exploratory; P3 did not establish a treatment-associated change.

Table S7. Prespecified analytical populations and sensitivity analyses.

Dataset	Analysis	Population	Decision/rationale
GSE10935	Primary	All 12 matched pairs	No sample excluded by the predefined two-metric rule
GSE10935	Sensitivity 1	GPL571 only; 10 pairs	Platform restriction
GSE10935	Sensitivity 2	Strict nine-pair subset	Excluded P01, P02, P09 for raw-intensity characteristics
GSE261589	Primary T/NK	4 RRP vs. 3 controls	All donors meeting structural requirements included
GSE261589	T/NK sensitivity	3 RRP vs. 3 controls	Excluded low-depth RRP1 by prespecified sensitivity rule
GSE261589	Primary myeloid	4 RRP vs. 3 controls	All donors meeting structural requirements included
GSE261589	Epithelial	Not analyzed between groups	Only one control donor met the predefined cell-count and sequencing-depth requirements
GSE275788	Primary	All 17 pairs	Week-47 pair included
GSE275788	Sensitivity 1: predefined quality-control subset	Predefined quality-control subset; 14 pairs	Excluded Pt1, Pt17, Pt26
GSE275788	Sensitivity 2	Week-52 only; 16 pairs	Excluded one Week-47 pair

Immune-program patterns across untreated RRP, exploratory single-cell context, and post-INO-3107 tissue

P1 — paired untreated tissue

Within-cohort reanalysis

CD8-associated cytotoxic program score ↓

NK-associated cytotoxic program score ↓

TGF-β immunosuppression program score ↑

versus autologous laryngeal mucosa

Same GSE10935/DeVoti cohort: recapitulation, not independent confirmation

P2/S2 — single-cell context

Exploratory only

No compartment-level program or broad cell class reached FDR < 0.05

Recovered T/NK fraction lower (nominal exact $p = 0.0286$; FDR $= 0.1286$)

Seven donors give coarse exact-permutation resolution; composition findings remain unresolved

HC1/HC2: <1% epithelial; HC3: epithelial-dominant — marked control heterogeneity

CD8/NK-associated program scores were not lower within recovered T/NK

Myeloid M2-like and TGF-β observations remain hypothesis-generating

P3 — paired post-treatment tissue

Within-dataset treatment-associated changes

CD8-associated cytotoxic program score ↑

NK-associated cytotoxic program score ↑

Th1/IFN-γ program score ↑

CXCR3-trafficking program score ↑

TGF-β estimate was not statistically distinguishable from zero; no conclusion about post-treatment change

Descriptive cross-dataset observation: CD8/NK-associated program changes have opposite signs in P1 and P3; no direct statistical test compared the two datasets.

The single-cell observations are hypothesis-generating and require matched laryngeal, spatial, functional, and response-linked validation. The framework does not establish causal mediation, complete immune normalization, a predictive biomarker, or a rationale for systemic TGF-β blockade.

Figure S1. Graphical summary of the main findings. The paired untreated-tissue and post-treatment analyses are shown separately from the exploratory single-cell findings. In P1, CD8-associated and NK-associated cytotoxic program scores were lower and the TGF-β immunosuppression program score was higher in papilloma than in matched autologous laryngeal mucosa. These findings reproduce the pattern previously reported from the same GSE10935 cohort and should not be considered independent confirmation. In P2/S2, no comparison remained significant after FDR correction, and these analyses are therefore shown only as exploratory cellular context. For S2, the seven-donor design also limits the resolution of exact permutation testing. In P3, CD8-associated, NK-associated, Th1/IFN-γ, and CXCR3-associated trafficking program scores increased after INO-3107. The P3 TGF-β estimate was not statistically distinguishable from zero, so these data do not establish a treatment-associated change in this program. The CD8- and NK-associated programs changed in opposite directions in P1 and P3; this is a descriptive cross-dataset observation and was not tested directly.
